# Supplementary material for: Investigating the potential of Juglans regia phytoconstituents for the treatment of cervical cancer utilizing network biology and molecular docking approach
Source: PLoS One. 2024 Apr 16;19(4):e0287864. doi: 10.1371/journal.pone.0287864 (PMC11020953; doi:10.1371/journal.pone.0287864)
Supplement: S1 File — R codes and documentation of complete Differential gene expression of data. (PDF) [file pone.0287864.s001.pdf]

S1: DEG analysis

#install packages

```
install.packages("BiocManager")
BiocManager::install("GEOquery")
BiocManager::install("DESeq2")
install.packages("forcast")
install.packages("stringr")
install.packages("ggrepel")
install.packages("readr")
install.packages("tidyr")
install.packages("survminer")
BiocManager::install("limma")
BiocManager::install("pheatmap")
```

#loading the packages

```
library(ggplot2)
library(ggrepel)
install.packages("magrittr") # package installations are only needed the first time you use it
install.packages("dplyr") # alternative installation of the %>%
library(magrittr) # needs to be run every time you start R and want to use %>%
library(dplyr)
library(ggplot2)
library(dplyr)
library(GEOquery)
library(limma)
```

## change my_id to be the dataset that you want.

my_id <- "GSE63678"

```
gse <- getGEO(my_id)
length(gse)
gse <- gse[[1]]gse
pData(gse) ## print the sample information
fData(gse) ## print the gene annotation
exprs(gse) ## print the expression data
## exprs get the expression levels as a data frame and get the distribution
summary(exprs(gse))
boxplot(exprs(gse), outline=FALSE)
```

#We take log 2 because values were too big and to make them in the lower range we use log 2 exprs(gse) <- log2(exprs(gse))

```
boxplot(exprs(gse),outline=FALSE)
View(exprs(gse))
sampleInfo <- pData(gse)
View(sampleInfo)
## source_name_ch1 and characteristics_ch1.1 seem to contain factors we might need for theanalysis. Let's
```

pick just those columns

```
sampleInfo <- select(sampleInfo, source_name_ch1,characteristics_ch1.1)##

Optionally, rename to more convenient column names

sampleInfo <- rename(sampleInfo,group = source_name_ch1, patient=characteristics_ch1.1)

View(sampleInfo)

library(pheatmap)

## argument use="c" stops an error if there are any missing data points
corMatrix <- cor(exprs(gse),use="c")

pheatmap(corMatrix) View(corMatrix)

## Print the row names of the sample information and check if it matches the correlation matrix

rownames(sampleInfo)

colnames(corMatrix)

library(ggplot2)

#imposing the matrix- very important

rownames(sampleInfo) <- colnames(corMatrix)

pheatmap(corMatrix,annotation_col=sampleInfo)

## MAKE SURE TO TRANSPOSE THE EXPRESSION MATRIX

pca <- prcomp(t(exprs(gse)))

## Join the PCs to the sample information

cbind(sampleInfo, pca$x) %>% ggplot(aes(x = PC1, y=PC2, col=group,label=paste("", patient))) +geom_point()
+ geom_text_repel()
design <- model.matrix(~0+sampleInfo$group)

View(design)

## the column names are a bit ugly, so we will rename

colnames(design) <- c( "cervical.cancer.tissue.cells","endometrial.cancer.tissue.cells","normal.endometrial.tissue.cells","nor
mal.cervical.tissue.cells","normal.vulvular.tissue.cells","vulvular.cancer.tissue.cells")

summary(exprs(gse))

View(exprs(gse))

## calculate median expression level

cutoff <- median(exprs(gse))

View(cutoff)

## TRUE or FALSE for whether each gene is "expressed" in each sample

is_expressed <- exprs(gse) > cutoff

## Identify genes expressed in more than 2 samples
keep <- rowSums(is_expressed) > 2
## check how many genes are removed / retained.

table(keep)

View(keep)

## subset to just those expressed genes

gse <- gse[keep,]

fit <- lmFit(exprs(gse), design)

head(fit$coefficients)

contrasts <-
```

```
makeContrasts(normal.cervical.tiss
ue.cells-
cervical.cancer.tissue.cells,normal.e
ndometrial.tissue.cells-
endometrial.cancer.tissue.cells,nor
mal.vulvular.tissue.cells-
vulvular.cancer.tissue.cells,
levels=design)

View(contrasts)
## can define multiple contrasts
## e.g. makeContrasts(Group1 - Group2, Group2 - Group3,............... levels=design)

fit2 <- contrasts.fit(fit, contrasts)fit2
<- eBayes(fit2,0.01)
tt <- topTable(fit2,adjust="fdr",sort.by="B" ,number=250)
topTable(fit2)
View(fit2) View(topTable(fit2,
coef=1))

#THIS GIVES LOGFC VALUES
View(decideTests(fit2))
View(table(decideTests(fit2)))
## calculate relative array weights
aw <- arrayWeights(exprs(gse),design)
View(aw)
fit <- lmFit(exprs(gse), design,weights = aw)
contrasts <- makeContrasts(normal.cervical.tissue.cells-
cervical.cancer.tissue.cells,normal.endometrial.tissue.cells-
endometrial.cancer.tissue.cells,normal.vulvular.tissue.cells-vulvular.cancer.tissue.cells,  levels=design)
fit2 <- contrasts.fit(fit, contrasts)fit2
<- eBayes(fit2)
View(fit2)
#
anno <- fData(gse)
View(anno)
colnames(anno) <- c( "ID","GBACC","SPOTID","SPECIES")
View(anno)
anno <- select(anno,ID,GBACC,SPOTID,SPECIES)
View(anno)
fit2$genes <- anno
View(topTable(fit2))
View(fit2)
full_results <- topTable(fit2, number=Inf)
```

library(ggplot2)

#using tibble library from tidyverse package

full_results <- tibble::rownames_to_column(full_results,"fc")

View(full_results)

ggplot(full_results,aes(x = logFC, y=B)) + geom_point()

## Create volcano plot

full_results <- topTable(fit2, coef=1, number=Inf)

library(ggplot2)

ggplot(full_results,aes(x = logFC, y=B)) + geom_point()##

change according to your needs

p_cutoff <- 0.05

fc_cutoff <- 1 #run

thsis full_results

%>%

mutate(Significant = adj.P.Val < p_cutoff, abs(logFC) > fc_cutoff) %>%ggplot(aes(x

= logFC, y = B, col=Significant)) + geom_point()

mutate(Significant = P.Value < p_cutoff, abs(logFC) > fc_cutoff) %>%

ggplot(aes(x = logFC, y = B, col=Significant)) + geom_point()

library(ggrepel) p_cutoff <- 0.05

fc_cutoff <- 1

topN <- 20

library(dplyr)

#error in the next line

#SYBMOL INSTEAD OF GBACC

full_results %>%

mutate(Significant = adj.P.Val < p_cutoff, abs(logFC) > fc_cutoff) %>%

mutate(Rank = 1:n(), Label = ifelse(Rank < topN, GBACC,"")) %>%

ggplot(aes(x = logFC, y = B, col=Significant,label=Label)) + geom_point() +
geom_text_repel(col="black")

topN <- 20

##

ids_of_interest <- mutate(full_results, Rank = 1:n()) %>% filter(Rank < topN) %>% pull(ID) gene_names <-

mutate(full_results, Rank = 1:n()) %>% filter(Rank < topN) %>% pull(SPOTID)gene_matrix <-

exprs(gse)[ids_of_interest,]

pheatmap(gene_matrix,labels_row = gene_names)

pheatmap(gene_matrix,labels_row = gene_names,scale="row")

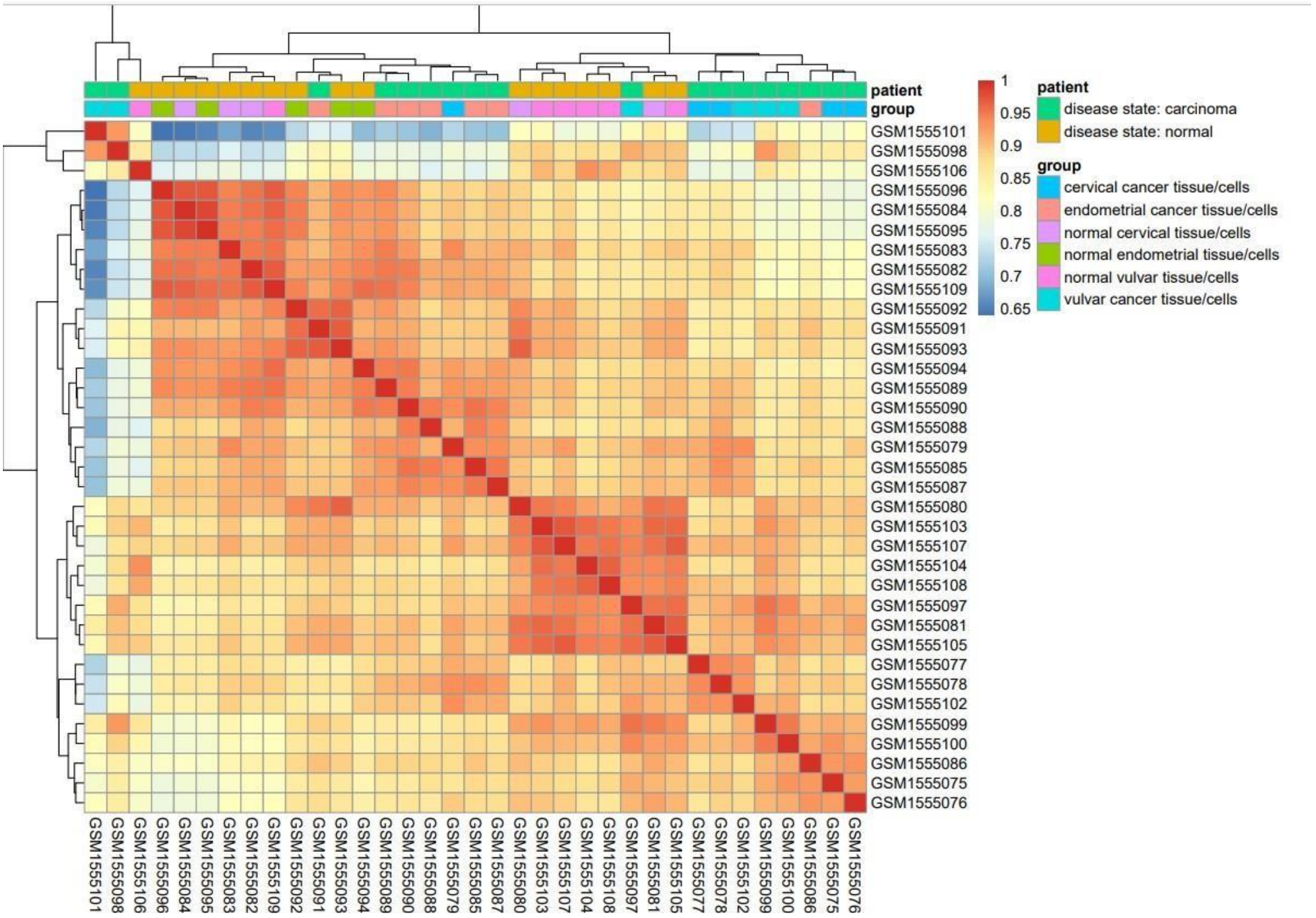

patient
group
GSM1555101
GSM1555098
GSM1555106
GSM1555096
GSM1555084
GSM1555095
GSM1555083
GSM1555082
GSM1555109
GSM1555092
GSM1555091
GSM1555093
GSM1555094
GSM1555089
GSM1555090
GSM1555088
GSM1555079
GSM1555085
GSM1555087
GSM1555080
GSM1555103
GSM1555107
GSM1555104
GSM1555108
GSM1555097
GSM1555081
GSM1555105
GSM1555077
GSM1555078
GSM1555102
GSM1555099
GSM1555100
GSM1555086
GSM1555075
GSM1555076
1
0.95
0.9
0.85
0.8
0.75
0.7
0.65
patient
disease state: carcinoma
disease state: normal
group
cervical cancer tissue/cells
endometrial cancer tissue/cells
normal cervical tissue/cells
normal endometrial tissue/cells
normal vulvar tissue/cells
vulvar cancer tissue/cells

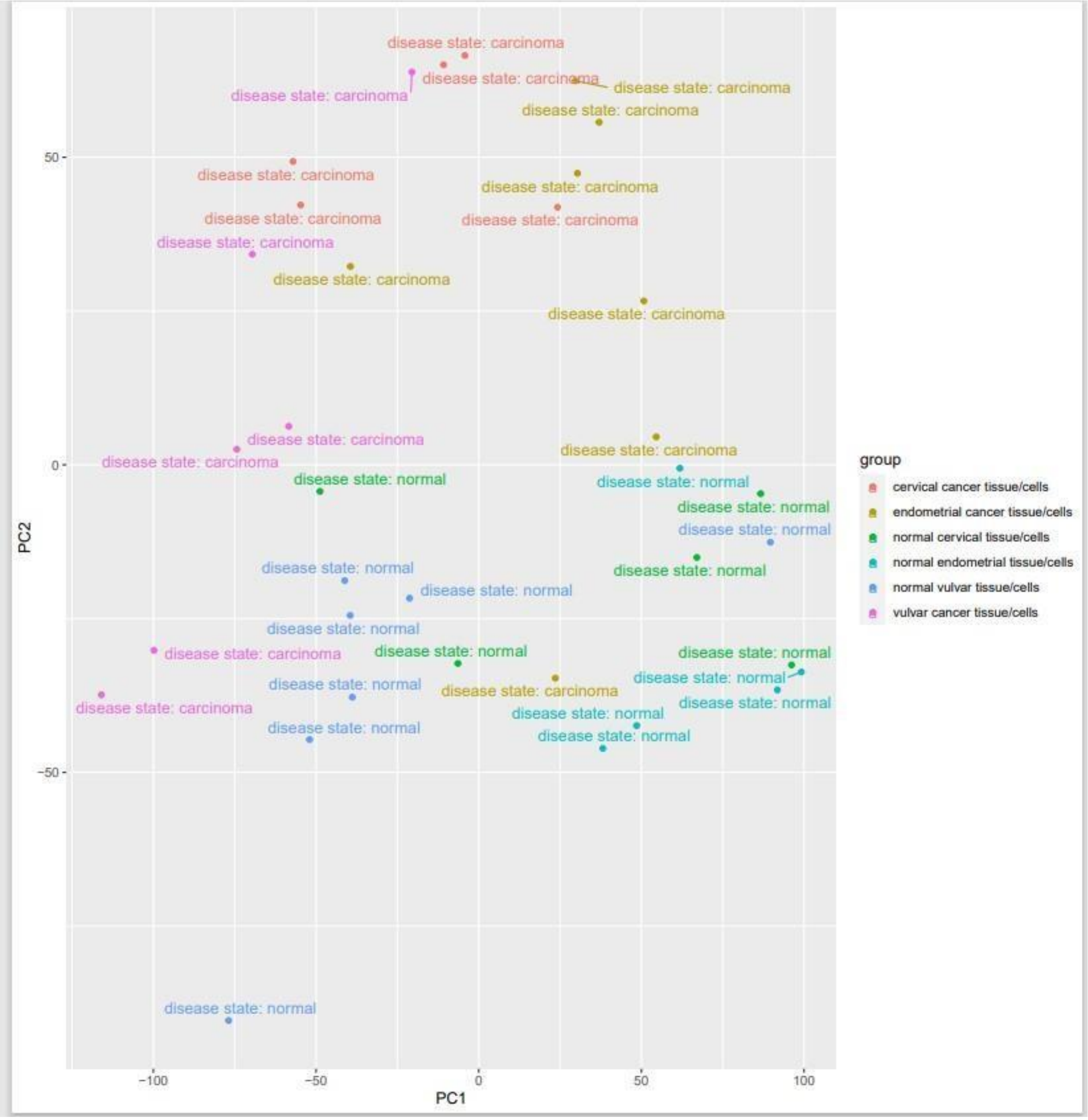

PC1
PC2
group
cervical cancer tissue/cells
endometrial cancer tissue/cells
normal cervical tissue/cells
normal endometrial tissue/cells
normal vulvar tissue/cells
vulvar cancer tissue/cells
disease state: carcinoma
disease state: normal

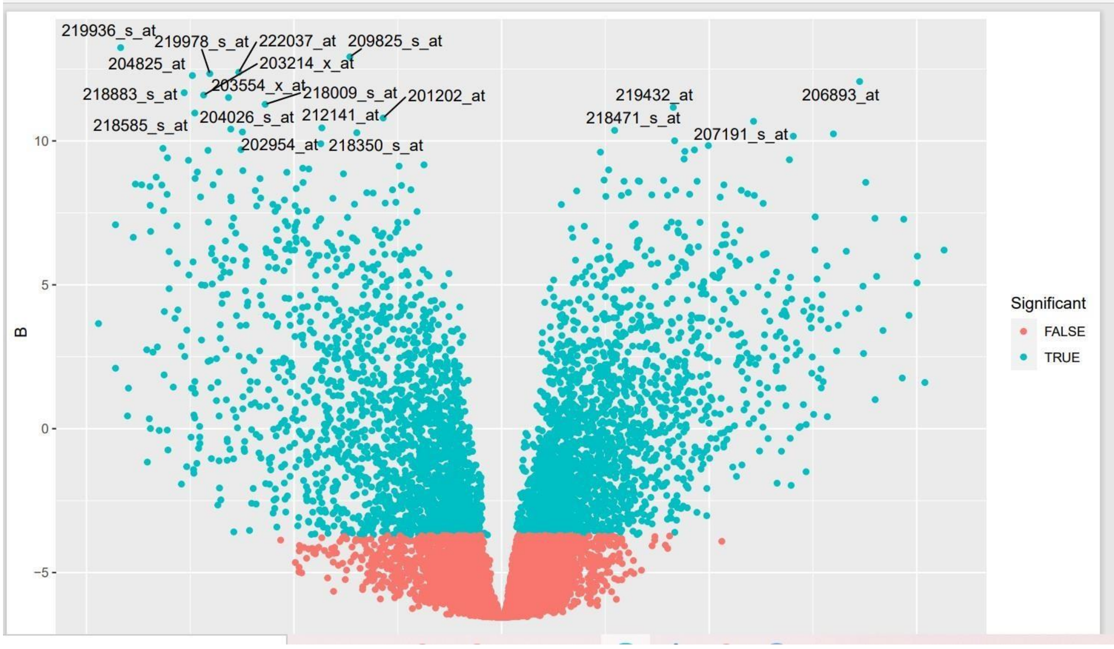

219936_s_at
219978_s_at
222037_at
209825_s_at
204825_at
203214_x_at
203554_x_at
218883_s_at
218009_s_at
201202_at
219432_at
206893_at
204026_s_at
212141_at
218471_s_at
218585_s_at
207191_s_at
202954_at
218350_s_at
10
5
0
−5
B
Significant
FALSE
TRUE

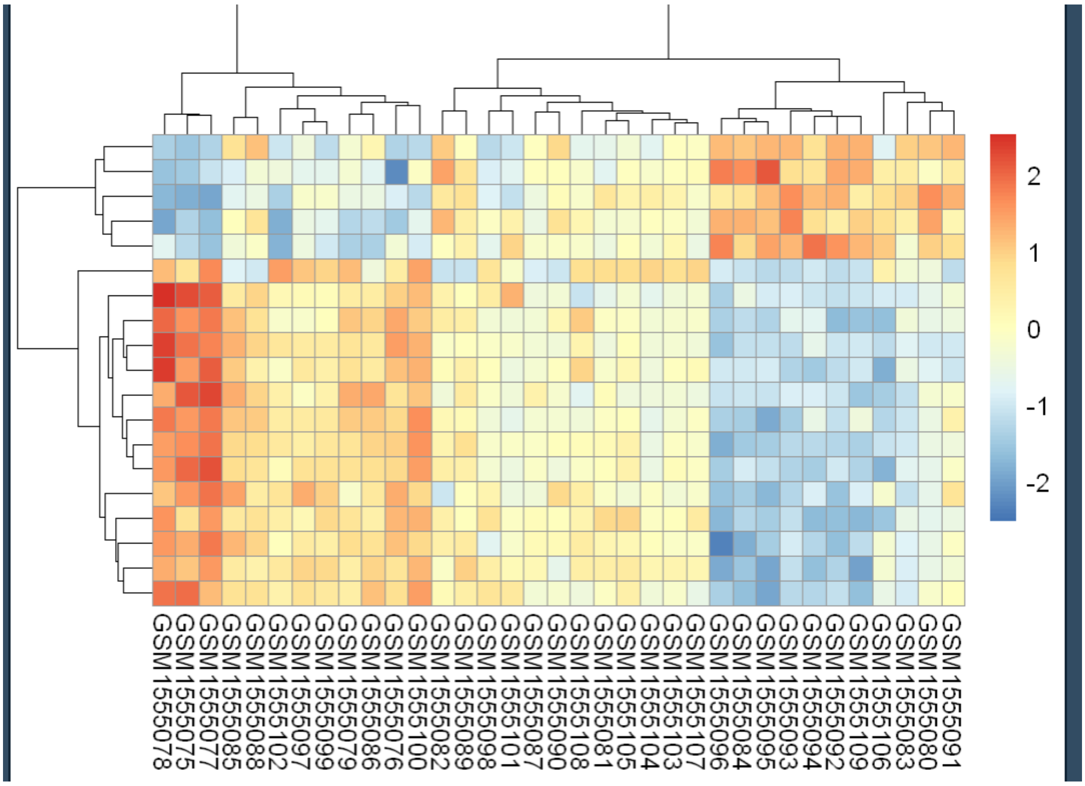

2
1
0
-1
-2
GSM1555078
GSM1555075
GSM1555077
GSM1555085
GSM1555088
GSM1555102
GSM1555097
GSM1555099
GSM1555079
GSM1555086
GSM1555076
GSM1555100
GSM1555082
GSM1555089
GSM1555098
GSM1555101
GSM1555087
GSM1555090
GSM1555108
GSM1555081
GSM1555105
GSM1555104
GSM1555103
GSM1555107
GSM1555096
GSM1555084
GSM1555095
GSM1555093
GSM1555094
GSM1555092
GSM1555109
GSM1555106
GSM1555083
GSM1555080
GSM1555091
