# Supplementary material for: Investigating the potential of Juglans regia phytoconstituents for the treatment of cervical cancer utilizing network biology and molecular docking approach
Source: PLoS One. 2024 Apr 16;19(4):e0287864. doi: 10.1371/journal.pone.0287864 (PMC11020953; doi:10.1371/journal.pone.0287864)
Supplement: S1 Table — 207 upregulated genes identified with Gene IDs and logFC values. (DOCX) [file pone.0287864.s002.docx]

**S1 Table: DEG identification**

| **ID** | **gene symbol** | **gene names** | **logFC** |
| --- | --- | --- | --- |
| 209242_at | PEG3 | Paternally expressed 3 | 1.068354 |
| 206067_s_at | WT1 | Wilms tumor 1 | 1.03805 |
| 219791_s_at | FLJ11539 | SAMSN1 | 0.982722 |
| 221950_at | EMX2 | empty spiracles homeobox 2 | 0.979774 |
| 204939_s_at | PLN | phospholamban | 0.972755 |
| 209613_s_at | ADH1B | alcohol dehydrogenase 1B (class I), beta polypeptide | 0.949736 |
| 206012_at | LEFTY2 | left-right determination factor 2 | 0.948617 |
| 209612_s_at | ADH1B |  | 0.921581 |
| 201496_x_at | MYH11 | myosin heavy chain 11 | 0.92024 |
| 213933_at | PTGER3 | Prostaglandin E receptor 3 | 0.895378 |
| 220272_at | BNC2 | Homo sapiens basonuclin 2 | 0.885717 |
| 206022_at | NDP-AS1 | NDP antisense RNA 1 | 0.875319 |
| 212843_at | NCAM1 | neural cell adhesion molecule 1 | 0.856023 |
| 209243_s_at | .. |  | 0.855918 |
| 202274_at | ACTG2 |  | 0.851954 |
| 211276_at | TCEAL2 | transcription elongation factor A like 2 | 0.849402 |
| 203951_at | CNN1 | calponin 1 | 0.845395 |
| 202222_s_at | DES | DESMIN | 0.840357 |
| 205358_at | GRIA2 | glutamate ionotropic receptor AMPA type subunit 2 | 0.835409 |
| 201497_x_at | MYH11 | myosin heavy chain 11 | 0.814122 |
| 206893_at | SALL1 | spalt like transcription factor 1 | 0.801172 |
| 204719_at | ABCA8 | ATP binding cassette subfamily A member 8 | 0.796486 |
| 218087_s_at | ORBS1 | sorbin and SH3 domain containing 1 | 0.796444 |
| 207016_s_at | ALDH1A2 | aldehyde dehydrogenase 1 family member A2 | 0.793168 |
| 218824_at | [PNMAL1](https://www.ncbi.nlm.nih.gov/geoprofiles?Db=gene&DbFrom=geoprofiles&Cmd=Link&LinkName=geoprofiles_gene&IdsFromResult=120283886) | paraneoplastic Ma antigen family like 1 | 0.791241 |
| 209763_at | CHRDL1 | chordin like 1 | 0.784044 |
| 216953_s_at | [WT1](https://www.ncbi.nlm.nih.gov/geoprofiles?Db=gene&DbFrom=geoprofiles&Cmd=Link&LinkName=geoprofiles_gene&IdsFromResult=100437644) | Wilms tumor 1 | 0.781398 |
| 212915_at | [PDZRN3](https://www.ncbi.nlm.nih.gov/geoprofiles?Db=gene&DbFrom=geoprofiles&Cmd=Link&LinkName=geoprofiles_gene&IdsFromResult=17847420) | PDZ domain containing ring finger 3 | 0.781002 |
| 212805_at | [PRUNE2](https://www.ncbi.nlm.nih.gov/geoprofiles?Db=gene&DbFrom=geoprofiles&Cmd=Link&LinkName=geoprofiles_gene&IdsFromResult=89125912) | prune homolog 2 | 0.778485 |
| 209948_at | [KCNMB1](https://www.ncbi.nlm.nih.gov/geoprofiles?Db=gene&DbFrom=geoprofiles&Cmd=Link&LinkName=geoprofiles_gene&IdsFromResult=51056555) | potassium calcium-activated channel subfamily M regulatory beta subunit 1 | 0.768269 |
| 219935_at | [ADAMTS5](https://www.ncbi.nlm.nih.gov/geoprofiles?Db=gene&DbFrom=geoprofiles&Cmd=Link&LinkName=geoprofiles_gene&IdsFromResult=12134099) | ADAM metallopeptidase with thrombospondin type 1 motif 5 | 0.756404 |
| 203766_s_at | [LMOD1](https://www.ncbi.nlm.nih.gov/geoprofiles?Db=gene&DbFrom=geoprofiles&Cmd=Link&LinkName=geoprofiles_gene&IdsFromResult=58584214) | leiomodin 1 | 0.754788 |
| 209687_at | [CXCL12](https://www.ncbi.nlm.nih.gov/geoprofiles?Db=gene&DbFrom=geoprofiles&Cmd=Link&LinkName=geoprofiles_gene&IdsFromResult=78109703) | C-X-C motif chemokine ligand 12 | 0.748842 |
| 205529_s_at | [RUNX1T1](https://www.ncbi.nlm.nih.gov/geoprofiles?Db=gene&DbFrom=geoprofiles&Cmd=Link&LinkName=geoprofiles_gene&IdsFromResult=123740246) | RUNX1 translocation partner 1 | 0.7481 |
| 204940_at | [PLN](https://www.ncbi.nlm.nih.gov/geoprofiles?Db=gene&DbFrom=geoprofiles&Cmd=Link&LinkName=geoprofiles_gene&IdsFromResult=65373366) | phospholamban | 0.73962 |
| 217897_at | [FXYD6](https://www.ncbi.nlm.nih.gov/geoprofiles?Db=gene&DbFrom=geoprofiles&Cmd=Link&LinkName=geoprofiles_gene&IdsFromResult=52917183) | FXYD domain containing ion transport regulator 6 | 0.737832 |
| 205433_at | [BCHE](https://www.ncbi.nlm.nih.gov/geoprofiles?Db=gene&DbFrom=geoprofiles&Cmd=Link&LinkName=geoprofiles_gene&IdsFromResult=91293305) | butyrylcholinesterase | 0.734799 |
| 218332_at | [BEX1](https://www.ncbi.nlm.nih.gov/geoprofiles?Db=gene&DbFrom=geoprofiles&Cmd=Link&LinkName=geoprofiles_gene&IdsFromResult=16305918) | brain expressed X-linked 1 | 0.73472 |
| 52837_at | [KIAA1644](https://www.ncbi.nlm.nih.gov/geoprofiles?Db=gene&DbFrom=geoprofiles&Cmd=Link&LinkName=geoprofiles_gene&IdsFromResult=102760983) |  | 0.734194 |
| 207789_s_at | [DPP6](https://www.ncbi.nlm.nih.gov/geoprofiles?Db=gene&DbFrom=geoprofiles&Cmd=Link&LinkName=geoprofiles_gene&IdsFromResult=18866431) | dipeptidyl peptidase like 6 | 0.726597 |
| 213823_at | [HOXA11](https://www.ncbi.nlm.nih.gov/geoprofiles?Db=gene&DbFrom=geoprofiles&Cmd=Link&LinkName=geoprofiles_gene&IdsFromResult=33575424) | homeobox A11 | 0.723997 |
| 204938_s_at | [PLN](https://www.ncbi.nlm.nih.gov/geoprofiles?Db=gene&DbFrom=geoprofiles&Cmd=Link&LinkName=geoprofiles_gene&IdsFromResult=65373366) | phospholamban | 0.720232 |
| 216333_x_at | [TNXB](https://www.ncbi.nlm.nih.gov/geoprofiles?Db=gene&DbFrom=geoprofiles&Cmd=Link&LinkName=geoprofiles_gene&IdsFromResult=33577926) | tenascin XB | 0.718633 |
| 204851_s_at | [DCX](https://www.ncbi.nlm.nih.gov/geoprofiles?Db=gene&DbFrom=geoprofiles&Cmd=Link&LinkName=geoprofiles_gene&IdsFromResult=66701278) | doublecortin | 0.712179 |
| 219778_at | [ZFPM2](https://www.ncbi.nlm.nih.gov/geoprofiles?Db=gene&DbFrom=geoprofiles&Cmd=Link&LinkName=geoprofiles_gene&IdsFromResult=101242863) | zinc finger protein, FOG family member 2 | 0.710818 |
| 205564_at | [PAGE4](https://www.ncbi.nlm.nih.gov/geoprofiles?Db=gene&DbFrom=geoprofiles&Cmd=Link&LinkName=geoprofiles_gene&IdsFromResult=54596691) | PAGE family member 4 | 0.709048 |
| 202409_at | [INS-IGF2](https://www.ncbi.nlm.nih.gov/geoprofiles?Db=gene&DbFrom=geoprofiles&Cmd=Link&LinkName=geoprofiles_gene&IdsFromResult=79062658) | [INS-IGF2 readthrough](https://www.ncbi.nlm.nih.gov/geoprofiles?Db=gene&DbFrom=geoprofiles&Cmd=Link&LinkName=geoprofiles_gene&IdsFromResult=79062658) | 0.70682 |
| 208131_s_at | [PTGIS](https://www.ncbi.nlm.nih.gov/geoprofiles?Db=gene&DbFrom=geoprofiles&Cmd=Link&LinkName=geoprofiles_gene&IdsFromResult=130148347) | prostaglandin I2 (prostacyclin) synthase | 0.704284 |
| 218934_s_at | [HSPB7](https://www.ncbi.nlm.nih.gov/geoprofiles?Db=gene&DbFrom=geoprofiles&Cmd=Link&LinkName=geoprofiles_gene&IdsFromResult=26567698) | heat shock protein family B (small) member 7 | 0.701545 |
| 218730_s_at | [OGN](https://www.ncbi.nlm.nih.gov/geoprofiles?Db=gene&DbFrom=geoprofiles&Cmd=Link&LinkName=geoprofiles_gene&IdsFromResult=125901579) | osteoglycin | 0.70153 |
| 204749_at | [NAP1L3](https://www.ncbi.nlm.nih.gov/geoprofiles?Db=gene&DbFrom=geoprofiles&Cmd=Link&LinkName=geoprofiles_gene&IdsFromResult=66701176) | nucleosome assembly protein 1 like 3 | 0.699344 |
| 221584_s_at | [KCNMA1](https://www.ncbi.nlm.nih.gov/geoprofiles?Db=gene&DbFrom=geoprofiles&Cmd=Link&LinkName=geoprofiles_gene&IdsFromResult=57804546) | potassium calcium-activated channel subfamily M alpha 1 | 0.69102 |
| 204422_s_at | [FGF2](https://www.ncbi.nlm.nih.gov/geoprofiles?Db=gene&DbFrom=geoprofiles&Cmd=Link&LinkName=geoprofiles_gene&IdsFromResult=108249470) | fibroblast growth factor 2 | 0.690185 |
| 219440_at | [RAI2](https://www.ncbi.nlm.nih.gov/geoprofiles?Db=gene&DbFrom=geoprofiles&Cmd=Link&LinkName=geoprofiles_gene&IdsFromResult=100440125) | retinoic acid induced 2 | 0.688331 |
| 213451_x_at | … |  | 0.685241 |
| 206167_s_at | ARHGAP6 |  | 0.685206 |
| 210299_s_at | [FHL1](https://www.ncbi.nlm.nih.gov/geoprofiles?Db=gene&DbFrom=geoprofiles&Cmd=Link&LinkName=geoprofiles_gene&IdsFromResult=100431100) | four and a half LIM domains 1 | 0.683844 |
| 213714_at | CACNB2 |  | 0.683482 |
| 203662_s_at | [TMOD1](https://www.ncbi.nlm.nih.gov/geoprofiles?Db=gene&DbFrom=geoprofiles&Cmd=Link&LinkName=geoprofiles_gene&IdsFromResult=71856598) | tropomodulin 1 | 0.681426 |
| 213316_at | [KIAA1462](https://www.ncbi.nlm.nih.gov/geoprofiles?Db=gene&DbFrom=geoprofiles&Cmd=Link&LinkName=geoprofiles_gene&IdsFromResult=78163018) |  | 0.680336 |
| 210832_x_at | [PTGER3](https://www.ncbi.nlm.nih.gov/geoprofiles?Db=gene&DbFrom=geoprofiles&Cmd=Link&LinkName=geoprofiles_gene&IdsFromResult=120823913) | prostaglandin E receptor 3 | 0.68031 |
| 213228_at | …. |  | 0.673026 |
| 214043_at | [PTPRD](https://www.ncbi.nlm.nih.gov/geoprofiles?Db=gene&DbFrom=geoprofiles&Cmd=Link&LinkName=geoprofiles_gene&IdsFromResult=93101743) | protein tyrosine phosphatase, receptor type D | 0.672876 |
| 204845_s_at | [ENPEP](https://www.ncbi.nlm.nih.gov/geoprofiles?Db=gene&DbFrom=geoprofiles&Cmd=Link&LinkName=geoprofiles_gene&IdsFromResult=3412672) | glutamyl aminopeptidase | 0.671385 |
| 221019_s_at | COLEC12 | collectin subfamily member 12 | 0.666505 |
| 204844_at | [ENPEP](https://www.ncbi.nlm.nih.gov/geoprofiles?Db=gene&DbFrom=geoprofiles&Cmd=Link&LinkName=geoprofiles_gene&IdsFromResult=110570392) | glutamyl aminopeptidase | 0.665062 |
| 205862_at | [GREB1](https://www.ncbi.nlm.nih.gov/geoprofiles?Db=gene&DbFrom=geoprofiles&Cmd=Link&LinkName=geoprofiles_gene&IdsFromResult=103367210) | growth regulation by estrogen in breast cancer 1 | 0.663977 |
| 206638_at | [HTR2B](https://www.ncbi.nlm.nih.gov/geoprofiles?Db=gene&DbFrom=geoprofiles&Cmd=Link&LinkName=geoprofiles_gene&IdsFromResult=12120964) | 5-hydroxytryptamine receptor 2B | 0.663321 |
| 222043_at | [CLU](https://app.uio.no/med/klinmed/correlation-browser/iliac-v1.1/index.php?var1=CLU) | clusterin | 0.663084 |
| 210374_x_at | [PTGER3](https://www.ncbi.nlm.nih.gov/geoprofiles?Db=gene&DbFrom=geoprofiles&Cmd=Link&LinkName=geoprofiles_gene&IdsFromResult=106742352) | prostaglandin E receptor 3 | 0.660697 |
| 206093_x_at | TNXA | tenascin XA (pseudogene) | 0.660475 |
| 203661_s_at | TMOD1 | tropomodulin 1 | 0.659865 |
| 207961_x_at | [MYH11](https://www.ncbi.nlm.nih.gov/geoprofiles?Db=gene&DbFrom=geoprofiles&Cmd=Link&LinkName=geoprofiles_gene&IdsFromResult=97345674) | myosin heavy chain 11 | 0.659248 |
| 202992_at | [C7](https://www.ncbi.nlm.nih.gov/geoprofiles?Db=gene&DbFrom=geoprofiles&Cmd=Link&LinkName=geoprofiles_gene&IdsFromResult=78126926) | complement component 7 | 0.658483 |
| 204793_at | [GPRASP1](https://www.ncbi.nlm.nih.gov/geoprofiles?Db=gene&DbFrom=geoprofiles&Cmd=Link&LinkName=geoprofiles_gene&IdsFromResult=27873520) | G protein-coupled receptor associated sorting protein 1 | 0.653814 |
| 214724_at | [DIXDC1](https://www.ncbi.nlm.nih.gov/geoprofiles?Db=gene&DbFrom=geoprofiles&Cmd=Link&LinkName=geoprofiles_gene&IdsFromResult=93102421) | DIX domain containing 1 | 0.651495 |
| 213568_at | OSR2 |  | 0.648669 |
| 206954_at | WT1-AS | WT1 antisense RNA | 0.647901 |
| 202965_s_at | [CAPN6](https://www.ncbi.nlm.nih.gov/geoprofiles?Db=gene&DbFrom=geoprofiles&Cmd=Link&LinkName=geoprofiles_gene&IdsFromResult=28782294) | calpain 6 | 0.646355 |
| 210375_at | [PTGER3](https://www.ncbi.nlm.nih.gov/geoprofiles?Db=gene&DbFrom=geoprofiles&Cmd=Link&LinkName=geoprofiles_gene&IdsFromResult=67871893) | prostaglandin E receptor 3 | 0.645993 |
| 205549_at | [PCP4](https://www.ncbi.nlm.nih.gov/geoprofiles?Db=gene&DbFrom=geoprofiles&Cmd=Link&LinkName=geoprofiles_gene&IdsFromResult=14711476) | Purkinje cell protein 4 | 0.645905 |
| 204894_s_at | [AOC3](https://www.ncbi.nlm.nih.gov/geoprofiles?Db=gene&DbFrom=geoprofiles&Cmd=Link&LinkName=geoprofiles_gene&IdsFromResult=89744449) | amine oxidase, copper containing 3 | 0.643788 |
| 210839_s_at | [ENPP2](https://www.ncbi.nlm.nih.gov/geoprofiles?Db=gene&DbFrom=geoprofiles&Cmd=Link&LinkName=geoprofiles_gene&IdsFromResult=6096199) | ectonucleotide pyrophosphatase/phosphodiesterase 2 | 0.641446 |
| 210170_at | [PDLIM3](https://www.ncbi.nlm.nih.gov/geoprofiles?Db=gene&DbFrom=geoprofiles&Cmd=Link&LinkName=geoprofiles_gene&IdsFromResult=66706554) | PDZ and LIM domain 3 | 0.641345 |
| 222161_at | [NAALAD2](https://www.ncbi.nlm.nih.gov/geoprofiles?Db=gene&DbFrom=geoprofiles&Cmd=Link&LinkName=geoprofiles_gene&IdsFromResult=28801321) | N-acetylated alpha-linked acidic dipeptidase 2 | 0.637044 |
| 205547_s_at | [TAGLN](https://www.ncbi.nlm.nih.gov/geoprofiles?Db=gene&DbFrom=geoprofiles&Cmd=Link&LinkName=geoprofiles_gene&IdsFromResult=85082595) | transgelin | 0.635447 |
| 201525_at | [APOD](https://www.ncbi.nlm.nih.gov/geoprofiles?Db=gene&DbFrom=geoprofiles&Cmd=Link&LinkName=geoprofiles_gene&IdsFromResult=125894222) | apolipoprotein D | 0.631168 |
| 202555_s_at | MYLK | myosin light chain kinase | 0.629193 |
| 209621_s_at | PDLIM3 | PDZ and LIM domain 3 | 0.627641 |
| 207191_s_at | ISLR | immunoglobulin superfamily containing leucine rich repeat | 0.627613 |
| 213904_at | FRRS1L | ferric chelate reductase 1 like | 0.626909 |
| 213438_at | [NFASC](https://www.ncbi.nlm.nih.gov/geoprofiles?Db=gene&DbFrom=geoprofiles&Cmd=Link&LinkName=geoprofiles_gene&IdsFromResult=27882019) | neurofascin | 0.626839 |
| 201540_at | [FHL1](https://www.ncbi.nlm.nih.gov/geoprofiles?Db=gene&DbFrom=geoprofiles&Cmd=Link&LinkName=geoprofiles_gene&IdsFromResult=97339268) | four and a half LIM domains 1 | 0.626772 |
| 220102_at | [FOXL2](https://www.ncbi.nlm.nih.gov/geoprofiles?Db=gene&DbFrom=geoprofiles&Cmd=Link&LinkName=geoprofiles_gene&IdsFromResult=101439187) | forkhead box L2 | 0.62377 |
| 201539_s_at | [FHL1](https://www.ncbi.nlm.nih.gov/geoprofiles?Db=gene&DbFrom=geoprofiles&Cmd=Link&LinkName=geoprofiles_gene&IdsFromResult=100422388) | four and a half LIM domains 1 | 0.621849 |
| 209541_at | [IGF1](https://app.uio.no/med/klinmed/correlation-browser/iliac-v1.1/index.php?var1=IGF1) |  | 0.619478 |
| 204154_at | [CDO1](https://www.ncbi.nlm.nih.gov/geoprofiles?Db=gene&DbFrom=geoprofiles&Cmd=Link&LinkName=geoprofiles_gene&IdsFromResult=124875781) | cysteine dioxygenase type 1 | 0.618642 |
| 202437_s_at | [CYP1B1](https://www.ncbi.nlm.nih.gov/geoprofiles?Db=gene&DbFrom=geoprofiles&Cmd=Link&LinkName=geoprofiles_gene&IdsFromResult=72060986) | cytochrome P450 family 1 subfamily B member 1 | 0.618547 |
| 201058_s_at | [MYL9](https://www.ncbi.nlm.nih.gov/geoprofiles?Db=gene&DbFrom=geoprofiles&Cmd=Link&LinkName=geoprofiles_gene&IdsFromResult=79061307) | myosin light chain 9 | 0.618122 |
| 209982_s_at | NRXN2 | eurexin 2 | 0.61666 |
| 202436_s_at | [CYP1B1](https://www.ncbi.nlm.nih.gov/geoprofiles?Db=gene&DbFrom=geoprofiles&Cmd=Link&LinkName=geoprofiles_gene&IdsFromResult=35214085) | cytochrome P450 family 1 subfamily B member 1 | 0.615727 |
| 209726_at | [A11](https://www.ncbi.nlm.nih.gov/geoprofiles?Db=gene&DbFrom=geoprofiles&Cmd=Link&LinkName=geoprofiles_gene&IdsFromResult=79069935) | carbonic anhydrase 11 | 0.613061 |
| 209656_s_at | [TMEM47](https://www.ncbi.nlm.nih.gov/geoprofiles?Db=gene&DbFrom=geoprofiles&Cmd=Link&LinkName=geoprofiles_gene&IdsFromResult=85086666) | transmembrane protein 47 | 0.612888 |
| 204051_s_at | [SFRP4](https://www.ncbi.nlm.nih.gov/geoprofiles?Db=gene&DbFrom=geoprofiles&Cmd=Link&LinkName=geoprofiles_gene&IdsFromResult=102463855) | secreted frizzled related protein 4 | 0.612644 |
| 203685_at | [BCL2](https://www.ncbi.nlm.nih.gov/geoprofiles?Db=gene&DbFrom=geoprofiles&Cmd=Link&LinkName=geoprofiles_gene&IdsFromResult=132096633) | BCL2, apoptosis regulato | 0.612313 |
| 219167_at | [RASL12](https://www.ncbi.nlm.nih.gov/geoprofiles?Db=gene&DbFrom=geoprofiles&Cmd=Link&LinkName=geoprofiles_gene&IdsFromResult=57501652) | RAS like family 12 | 0.611915 |
| 210831_s_at | [PTGER3](https://www.ncbi.nlm.nih.gov/geoprofiles?Db=gene&DbFrom=geoprofiles&Cmd=Link&LinkName=geoprofiles_gene&IdsFromResult=100305712) | prostaglandin E receptor 3 | 0.610933 |
| 204041_at | [MAOB](https://www.ncbi.nlm.nih.gov/geoprofiles?Db=gene&DbFrom=geoprofiles&Cmd=Link&LinkName=geoprofiles_gene&IdsFromResult=77346389) | monoamine oxidase B | 0.60798 |
| 208609_s_at | [TNXB](https://www.ncbi.nlm.nih.gov/geoprofiles?Db=gene&DbFrom=geoprofiles&Cmd=Link&LinkName=geoprofiles_gene&IdsFromResult=130154352) | tenascin XB | 0.607451 |
| 204424_s_at | [LMO3](https://www.ncbi.nlm.nih.gov/geoprofiles?Db=gene&DbFrom=geoprofiles&Cmd=Link&LinkName=geoprofiles_gene&IdsFromResult=36033405) | LIM domain only 3 | 0.606359 |
| 214505_s_at | [FHL1](https://www.ncbi.nlm.nih.gov/geoprofiles?Db=gene&DbFrom=geoprofiles&Cmd=Link&LinkName=geoprofiles_gene&IdsFromResult=52913805) | four and a half LIM domains 1 | 0.603944 |
| 205522_at | [HOXD4](https://www.ncbi.nlm.nih.gov/geoprofiles?Db=gene&DbFrom=geoprofiles&Cmd=Link&LinkName=geoprofiles_gene&IdsFromResult=79494270) | homeobox D4 | 0.602599 |
| 205528_s_at | [RUNX1T1](https://www.ncbi.nlm.nih.gov/geoprofiles?Db=gene&DbFrom=geoprofiles&Cmd=Link&LinkName=geoprofiles_gene&IdsFromResult=22061576) | RUNX1 translocation partner 1 | 0.597984 |
| 206084_at | [PTPRR](https://www.ncbi.nlm.nih.gov/geoprofiles?Db=gene&DbFrom=geoprofiles&Cmd=Link&LinkName=geoprofiles_gene&IdsFromResult=102722031) | protein tyrosine phosphatase, receptor type R | 0.595119 |
| 220817_at | [TRPC4](https://www.ncbi.nlm.nih.gov/geoprofiles?Db=gene&DbFrom=geoprofiles&Cmd=Link&LinkName=geoprofiles_gene&IdsFromResult=61198202) | transient receptor potential cation channel subfamily C member 4 | 0.593579 |
| 202435_s_at | [CYP1B1](https://www.ncbi.nlm.nih.gov/geoprofiles?Db=gene&DbFrom=geoprofiles&Cmd=Link&LinkName=geoprofiles_gene&IdsFromResult=85079484) | cytochrome P450 family 1 subfamily B member 1 | 0.592334 |
| 204688_at | [SGCE](https://www.ncbi.nlm.nih.gov/geoprofiles?Db=gene&DbFrom=geoprofiles&Cmd=Link&LinkName=geoprofiles_gene&IdsFromResult=78130682) | sarcoglycan epsilon | 0.591698 |
| 205648_at | [WNT2](https://www.ncbi.nlm.nih.gov/geoprofiles?Db=gene&DbFrom=geoprofiles&Cmd=Link&LinkName=geoprofiles_gene&IdsFromResult=110571196) | Wnt family member 2 | 0.591566 |
| 219902_at | [BHMT2](https://www.ncbi.nlm.nih.gov/geoprofiles?Db=gene&DbFrom=geoprofiles&Cmd=Link&LinkName=geoprofiles_gene&IdsFromResult=65986266) | betaine--homocysteine S-methyltransferase 2 | 0.591368 |
| 214078_at | [PAK3](https://www.ncbi.nlm.nih.gov/geoprofiles?Db=gene&DbFrom=geoprofiles&Cmd=Link&LinkName=geoprofiles_gene&IdsFromResult=125054278) | p21 (RAC1) activated kinase 3 | 0.59072 |
| 208491_s_at | [PGM5](https://www.ncbi.nlm.nih.gov/geoprofiles?Db=gene&DbFrom=geoprofiles&Cmd=Link&LinkName=geoprofiles_gene&IdsFromResult=123752201) | phosphoglucomutase 5 | 0.590581 |
| 213413_at | [STON1](https://www.ncbi.nlm.nih.gov/geoprofiles?Db=gene&DbFrom=geoprofiles&Cmd=Link&LinkName=geoprofiles_gene&IdsFromResult=104239815) | stonin 1 | 0.58991 |
| 222101_s_at | [DCHS1](https://www.ncbi.nlm.nih.gov/geoprofiles?Db=gene&DbFrom=geoprofiles&Cmd=Link&LinkName=geoprofiles_gene&IdsFromResult=109007682) | dachsous cadherin-related 1 | 0.589245 |
| 213745_at | [ATRNL1](https://www.ncbi.nlm.nih.gov/geoprofiles?Db=gene&DbFrom=geoprofiles&Cmd=Link&LinkName=geoprofiles_gene&IdsFromResult=66837446) | attractin like 1 | 0.588837 |
| 204052_s_at | [SFRP4](https://www.ncbi.nlm.nih.gov/geoprofiles?Db=gene&DbFrom=geoprofiles&Cmd=Link&LinkName=geoprofiles_gene&IdsFromResult=102463855) | secreted frizzled related protein 4 | 0.586008 |
| 209392_at | [ENPP2](https://www.ncbi.nlm.nih.gov/geoprofiles?Db=gene&DbFrom=geoprofiles&Cmd=Link&LinkName=geoprofiles_gene&IdsFromResult=93097206) | ectonucleotide pyrophosphatase/phosphodiesterase 2 | 0.584404 |
| 220518_at | [ABI3BP](https://www.ncbi.nlm.nih.gov/geoprofiles?Db=gene&DbFrom=geoprofiles&Cmd=Link&LinkName=geoprofiles_gene&IdsFromResult=13694082) | ABI family member 3 binding protein | 0.582036 |
| 218162_at | OLFML3 | olfactomedin like 3 | 0.580608 |
| 205110_s_at | [FGF13](https://www.ncbi.nlm.nih.gov/geoprofiles?Db=gene&DbFrom=geoprofiles&Cmd=Link&LinkName=geoprofiles_gene&IdsFromResult=88843458) | fibroblast growth factor 13 | 0.580055 |
| 208792_s_at | [CLU](https://www.ncbi.nlm.nih.gov/geoprofiles?Db=gene&DbFrom=geoprofiles&Cmd=Link&LinkName=geoprofiles_gene&IdsFromResult=106062307) | clusterin | 0.576237 |
| 205225_at | [ESR1](https://www.ncbi.nlm.nih.gov/geoprofiles?Db=gene&DbFrom=geoprofiles&Cmd=Link&LinkName=geoprofiles_gene&IdsFromResult=77632273) | estrogen receptor 1 | 0.576221 |
| 213122_at | [TSPYL5](https://www.ncbi.nlm.nih.gov/geoprofiles?Db=gene&DbFrom=geoprofiles&Cmd=Link&LinkName=geoprofiles_gene&IdsFromResult=77640026) | TSPY like 5 | 0.576085 |
| 215073_s_at | [NR2F2](https://www.ncbi.nlm.nih.gov/geoprofiles?Db=gene&DbFrom=geoprofiles&Cmd=Link&LinkName=geoprofiles_gene&IdsFromResult=77357268) | nuclear receptor subfamily 2 group F member 2 | 0.575935 |
| 208791_at | [CLU](https://www.ncbi.nlm.nih.gov/geoprofiles?Db=gene&DbFrom=geoprofiles&Cmd=Link&LinkName=geoprofiles_gene&IdsFromResult=106062307) | clusterin | 0.575154 |
| 203903_s_at | [HEPH](https://www.ncbi.nlm.nih.gov/geoprofiles?Db=gene&DbFrom=geoprofiles&Cmd=Link&LinkName=geoprofiles_gene&IdsFromResult=85566451) | hephaestin | 0.57363 |
| 218694_at | ARMCX1 | armadillo repeat containing X-linked 1 | 0.572818 |
| 202920_at | [ANK2](https://www.ncbi.nlm.nih.gov/geoprofiles?Db=gene&DbFrom=geoprofiles&Cmd=Link&LinkName=geoprofiles_gene&IdsFromResult=66699349) | ankyrin 2, neuronal | 0.571673 |
| 204850_s_at | [DCX](https://www.ncbi.nlm.nih.gov/geoprofiles?Db=gene&DbFrom=geoprofiles&Cmd=Link&LinkName=geoprofiles_gene&IdsFromResult=66701278) | doublecortin | 0.571335 |
| 209209_s_at | [FERMT2](https://app.uio.no/med/klinmed/correlation-browser/iliac-v1.1/index.php?var1=FERMT2) |  | 0.569843 |
| 204457_s_at | [GAS1](https://www.ncbi.nlm.nih.gov/geoprofiles?Db=gene&DbFrom=geoprofiles&Cmd=Link&LinkName=geoprofiles_gene&IdsFromResult=12118784) | growth arrest specific 1 | 0.568835 |
| 219871_at | KLF3-AS1 | KLF3 antisense RNA 1 | 0.566992 |
| 212686_at | [PPM1H](https://www.ncbi.nlm.nih.gov/geoprofiles?Db=gene&DbFrom=geoprofiles&Cmd=Link&LinkName=geoprofiles_gene&IdsFromResult=108257592) | protein phosphatase, Mg2+/Mn2+ dependent 1H | 0.565832 |
| 210675_s_at | [PTPRR](https://www.ncbi.nlm.nih.gov/geoprofiles?Db=gene&DbFrom=geoprofiles&Cmd=Link&LinkName=geoprofiles_gene&IdsFromResult=102726560) | protein tyrosine phosphatase, receptor type R | 0.563944 |
| 219511_s_at | [SNCAIP](https://www.ncbi.nlm.nih.gov/geoprofiles?Db=gene&DbFrom=geoprofiles&Cmd=Link&LinkName=geoprofiles_gene&IdsFromResult=104245896) | synuclein alpha interacting protein | 0.562393 |
| 203999_at | [SYT1](https://www.ncbi.nlm.nih.gov/geoprofiles?Db=gene&DbFrom=geoprofiles&Cmd=Link&LinkName=geoprofiles_gene&IdsFromResult=67869921) | synaptotagmin 1 | 0.56218 |
| 213992_at | [COL4A6](https://www.ncbi.nlm.nih.gov/geoprofiles?Db=gene&DbFrom=geoprofiles&Cmd=Link&LinkName=geoprofiles_gene&IdsFromResult=58594292) | collagen type IV alpha 6 chain | 0.560237 |
| 212813_at | [JAM3](https://www.ncbi.nlm.nih.gov/geoprofiles?Db=gene&DbFrom=geoprofiles&Cmd=Link&LinkName=geoprofiles_gene&IdsFromResult=85089719) | junctional adhesion molecule 3 | 0.5601 |
| 206481_s_at | [LDB2](https://www.ncbi.nlm.nih.gov/geoprofiles?Db=gene&DbFrom=geoprofiles&Cmd=Link&LinkName=geoprofiles_gene&IdsFromResult=85083528) | LIM domain binding 2 | 0.557183 |
| 220906_at | .. |  | 0.553402 |
| 216081_at | [LAMA4](https://www.ncbi.nlm.nih.gov/geoprofiles?Db=gene&DbFrom=geoprofiles&Cmd=Link&LinkName=geoprofiles_gene&IdsFromResult=81547274) | laminin subunit alpha 4 | 0.552446 |
| 213249_at | [FBXL7](https://www.ncbi.nlm.nih.gov/geoprofiles?Db=gene&DbFrom=geoprofiles&Cmd=Link&LinkName=geoprofiles_gene&IdsFromResult=59184952) | F-box and leucine rich repeat protein 7 | 0.550407 |
| 203666_at | [CXCL12](https://www.ncbi.nlm.nih.gov/geoprofiles?Db=gene&DbFrom=geoprofiles&Cmd=Link&LinkName=geoprofiles_gene&IdsFromResult=78109703) | C-X-C motif chemokine ligand 12 | 0.549539 |
| 219637_at | [ARMC9](https://www.ncbi.nlm.nih.gov/geoprofiles?Db=gene&DbFrom=geoprofiles&Cmd=Link&LinkName=geoprofiles_gene&IdsFromResult=130146638) | armadillo repeat containing 9 | 0.549512 |
| 209894_at | [LEPROT](https://www.ncbi.nlm.nih.gov/geoprofiles?Db=gene&DbFrom=geoprofiles&Cmd=Link&LinkName=geoprofiles_gene&IdsFromResult=30546272) | eptin receptor overlapping transcript | 0.549043 |
| 220765_s_at | [LIMS2](https://www.ncbi.nlm.nih.gov/geoprofiles?Db=gene&DbFrom=geoprofiles&Cmd=Link&LinkName=geoprofiles_gene&IdsFromResult=58601050) | LIM zinc finger domain containing 2 | 0.548243 |
| 209540_at | [IGF1](https://www.ncbi.nlm.nih.gov/geoprofiles?Db=gene&DbFrom=geoprofiles&Cmd=Link&LinkName=geoprofiles_gene&IdsFromResult=78113027) | insulin like growth factor 1 | 0.546985 |
| 204163_at | [EMILIN1](https://www.ncbi.nlm.nih.gov/geoprofiles?Db=gene&DbFrom=geoprofiles&Cmd=Link&LinkName=geoprofiles_gene&IdsFromResult=132097111) | elastin microfibril interfacer 1 | 0.546666 |
| 213943_at | [TWIST1](https://www.ncbi.nlm.nih.gov/geoprofiles?Db=gene&DbFrom=geoprofiles&Cmd=Link&LinkName=geoprofiles_gene&IdsFromResult=59185643) | twist family bHLH transcription factor 1 | 0.544452 |
| 218312_s_at | [ZSCAN18](https://www.ncbi.nlm.nih.gov/geoprofiles?Db=gene&DbFrom=geoprofiles&Cmd=Link&LinkName=geoprofiles_gene&IdsFromResult=108263198) | zinc finger and SCAN domain containing 18 | 0.54228 |
| 219949_at | [LRRC2](https://www.ncbi.nlm.nih.gov/geoprofiles?Db=gene&DbFrom=geoprofiles&Cmd=Link&LinkName=geoprofiles_gene&IdsFromResult=97311034) | leucine rich repeat containing 2 | 0.541159 |
| 205392_s_at | CCL14 | C-C motif chemokine ligand 14 | 0.541024 |
| 214027_x_at | FAM48A | family with sequence similarity 48, member A | 0.540482 |
| 208305_at | PGR | progesterone receptor | 0.539317 |
| 211700_s_at | TRO | trophinin | 0.53703 |
| 206306_at | RYR3 | ryanodine receptor 3 | 0.536878 |
| 213946_s_at | .. |  | 0.535629 |
| 219087_at | [ASPN](https://www.ncbi.nlm.nih.gov/geoprofiles?Db=gene&DbFrom=geoprofiles&Cmd=Link&LinkName=geoprofiles_gene&IdsFromResult=57894451) | asporin | 0.534766 |
| 219682_s_at | [TBX3](https://www.ncbi.nlm.nih.gov/geoprofiles?Db=gene&DbFrom=geoprofiles&Cmd=Link&LinkName=geoprofiles_gene&IdsFromResult=57802646) | T-box 3 | 0.534606 |
| 220794_at | [GREM2](https://www.ncbi.nlm.nih.gov/geoprofiles?Db=gene&DbFrom=geoprofiles&Cmd=Link&LinkName=geoprofiles_gene&IdsFromResult=74874658) | gremlin 2, DAN family BMP antagonist | 0.534554 |
| 221814_at | [ADGRA2](https://www.ncbi.nlm.nih.gov/geoprofiles?Db=gene&DbFrom=geoprofiles&Cmd=Link&LinkName=geoprofiles_gene&IdsFromResult=43468695) | adhesion G protein-coupled receptor A2 | 0.534467 |
| 204955_at | [SRPX](https://www.ncbi.nlm.nih.gov/geoprofiles?Db=gene&DbFrom=geoprofiles&Cmd=Link&LinkName=geoprofiles_gene&IdsFromResult=78134042) | sushi repeat containing protein, X-linked | 0.53399 |
| 219636_s_at | [ARMC9](https://www.ncbi.nlm.nih.gov/geoprofiles?Db=gene&DbFrom=geoprofiles&Cmd=Link&LinkName=geoprofiles_gene&IdsFromResult=130146638) | armadillo repeat containing 9 | 0.533858 |
| 211110_s_at | [AR](https://www.ncbi.nlm.nih.gov/geoprofiles?Db=gene&DbFrom=geoprofiles&Cmd=Link&LinkName=geoprofiles_gene&IdsFromResult=83374375) | Androgen receptor | 0.531457 |
| 205303_at | [KCNJ8](https://www.ncbi.nlm.nih.gov/geoprofiles?Db=gene&DbFrom=geoprofiles&Cmd=Link&LinkName=geoprofiles_gene&IdsFromResult=98834551) | potassium voltage-gated channel subfamily J member 8 | 0.530728 |
| 217617_at | .. |  | 0.530049 |
| 213150_at | [HOXA10](https://www.ncbi.nlm.nih.gov/geoprofiles?Db=gene&DbFrom=geoprofiles&Cmd=Link&LinkName=geoprofiles_gene&IdsFromResult=93913233) | homeobox A10 | 0.528691 |
| 219370_at | [RPRM](https://www.ncbi.nlm.nih.gov/geoprofiles?Db=gene&DbFrom=geoprofiles&Cmd=Link&LinkName=geoprofiles_gene&IdsFromResult=110584755) | TP53 dependent G2 arrest mediator candidate | 0.527676 |
| 209616_s_at | [LOC100653057](https://www.ncbi.nlm.nih.gov/geoprofiles?Db=gene&DbFrom=geoprofiles&Cmd=Link&LinkName=geoprofiles_gene&IdsFromResult=95226027) | liver carboxylesterase 1-like | 0.527429 |
| 212764_at | .. |  | 0.526824 |
| 220116_at | [KCNN2](https://www.ncbi.nlm.nih.gov/geoprofiles?Db=gene&DbFrom=geoprofiles&Cmd=Link&LinkName=geoprofiles_gene&IdsFromResult=81551301) | potassium calcium-activated channel subfamily N member 2 | 0.526178 |
| 202202_s_at | [LAMA4](https://www.ncbi.nlm.nih.gov/geoprofiles?Db=gene&DbFrom=geoprofiles&Cmd=Link&LinkName=geoprofiles_gene&IdsFromResult=78708295) | laminin subunit alpha 4 | 0.525425 |
| 202508_s_at | [SNAP25](https://www.ncbi.nlm.nih.gov/geoprofiles?Db=gene&DbFrom=geoprofiles&Cmd=Link&LinkName=geoprofiles_gene&IdsFromResult=66292657) | synaptosome associated protein 25 | 0.523634 |
| 205883_at | [ZBTB16](https://www.ncbi.nlm.nih.gov/geoprofiles?Db=gene&DbFrom=geoprofiles&Cmd=Link&LinkName=geoprofiles_gene&IdsFromResult=27874610) | zinc finger and BTB domain containing 16 | 0.522965 |
| 213488_at | [SNED1](https://www.ncbi.nlm.nih.gov/geoprofiles?Db=gene&DbFrom=geoprofiles&Cmd=Link&LinkName=geoprofiles_gene&IdsFromResult=110578890) | sushi, nidogen and EGF like domains 1 | 0.522095 |
| 205381_at | [LRRC17](https://www.ncbi.nlm.nih.gov/geoprofiles?Db=gene&DbFrom=geoprofiles&Cmd=Link&LinkName=geoprofiles_gene&IdsFromResult=111039336) | leucine rich repeat containing 17 | 0.520889 |
| 211562_s_at | [LMOD1](https://www.ncbi.nlm.nih.gov/geoprofiles?Db=gene&DbFrom=geoprofiles&Cmd=Link&LinkName=geoprofiles_gene&IdsFromResult=89124688) | leiomodin 1 | 0.519176 |
| 219685_at | [TMEM35A](https://www.ncbi.nlm.nih.gov/geoprofiles?Db=gene&DbFrom=geoprofiles&Cmd=Link&LinkName=geoprofiles_gene&IdsFromResult=66843370) | transmembrane protein 35A | 0.518461 |
| 205304_s_at | [KCNJ8](https://www.ncbi.nlm.nih.gov/geoprofiles?Db=gene&DbFrom=geoprofiles&Cmd=Link&LinkName=geoprofiles_gene&IdsFromResult=98834551) | potassium voltage-gated channel subfamily J member 8 | 0.517359 |
| 204731_at | [TGFBR3](https://www.ncbi.nlm.nih.gov/geoprofiles?Db=gene&DbFrom=geoprofiles&Cmd=Link&LinkName=geoprofiles_gene&IdsFromResult=103422479) | transforming growth factor beta receptor 3 | 0.516247 |
| 221207_s_at | [NBEA](https://www.ncbi.nlm.nih.gov/geoprofiles?Db=gene&DbFrom=geoprofiles&Cmd=Link&LinkName=geoprofiles_gene&IdsFromResult=53158691) | neurobeachin | 0.516053 |
| 203088_at | FBLN5 | fibulin 5 | 0.515832 |
| 211006_s_at | [KCNB1](https://www.ncbi.nlm.nih.gov/geoprofiles?Db=gene&DbFrom=geoprofiles&Cmd=Link&LinkName=geoprofiles_gene&IdsFromResult=79071174) | potassium voltage-gated channel subfamily B member 1 | 0.515659 |
| 207390_s_at | [SMTN](https://www.ncbi.nlm.nih.gov/geoprofiles?Db=gene&DbFrom=geoprofiles&Cmd=Link&LinkName=geoprofiles_gene&IdsFromResult=12121713) | smoothelin | 0.514195 |
| 205880_at | [PRKD1](https://www.ncbi.nlm.nih.gov/geoprofiles?Db=gene&DbFrom=geoprofiles&Cmd=Link&LinkName=geoprofiles_gene&IdsFromResult=58586328) | protein kinase D1 | 0.513165 |
| 203304_at | [BAMBI](https://www.ncbi.nlm.nih.gov/geoprofiles?Db=gene&DbFrom=geoprofiles&Cmd=Link&LinkName=geoprofiles_gene&IdsFromResult=95219752) | BMP and activin membrane bound inhibitor | 0.511996 |
| 215555_at | CDNA |  | 0.511736 |
| 205803_s_at | [TRPC1](https://www.ncbi.nlm.nih.gov/geoprofiles?Db=gene&DbFrom=geoprofiles&Cmd=Link&LinkName=geoprofiles_gene&IdsFromResult=66295950) | transient receptor potential cation channel subfamily C member 1 | 0.509643 |
| 209343_at | [EFHD1](https://www.ncbi.nlm.nih.gov/geoprofiles?Db=gene&DbFrom=geoprofiles&Cmd=Link&LinkName=geoprofiles_gene&IdsFromResult=89748652) | EF-hand domain family member D1 | 0.508504 |
| 205066_s_at | [ENPP1](https://www.ncbi.nlm.nih.gov/geoprofiles?Db=gene&DbFrom=geoprofiles&Cmd=Link&LinkName=geoprofiles_gene&IdsFromResult=77912201) | ectonucleotide pyrophosphatase/phosphodiesterase 1 | 0.507766 |
| 203131_at | [PDGFRA](https://www.ncbi.nlm.nih.gov/geoprofiles?Db=gene&DbFrom=geoprofiles&Cmd=Link&LinkName=geoprofiles_gene&IdsFromResult=78125832) | platelet derived growth factor receptor alpha | 0.507142 |
| 209708_at | [MOXD1](https://www.ncbi.nlm.nih.gov/geoprofiles?Db=gene&DbFrom=geoprofiles&Cmd=Link&LinkName=geoprofiles_gene&IdsFromResult=112051248) | monooxygenase DBH like 1 | 0.506854 |
| 209447_at | [SYNE1](https://www.ncbi.nlm.nih.gov/geoprofiles?Db=gene&DbFrom=geoprofiles&Cmd=Link&LinkName=geoprofiles_gene&IdsFromResult=125049761) | spectrin repeat containing nuclear envelope protein 1 | 0.505977 |
| 213844_at | [HOXA5](https://www.ncbi.nlm.nih.gov/geoprofiles?Db=gene&DbFrom=geoprofiles&Cmd=Link&LinkName=geoprofiles_gene&IdsFromResult=54445745) | homeobox A5 | 0.50551 |
| 209735_at | [ABCG2](https://www.ncbi.nlm.nih.gov/geoprofiles?Db=gene&DbFrom=geoprofiles&Cmd=Link&LinkName=geoprofiles_gene&IdsFromResult=97300944) | ATP binding cassette subfamily G member 2 | 0.504063 |
| 209335_at | [DCN](https://www.ncbi.nlm.nih.gov/geoprofiles?Db=gene&DbFrom=geoprofiles&Cmd=Link&LinkName=geoprofiles_gene&IdsFromResult=78112977) | decorin | 0.503433 |
| 214212_x_at | [FERMT2](https://app.uio.no/med/klinmed/correlation-browser/iliac-v1.1/index.php?var1=FERMT2) |  | 0.501707 |
